# Non-Rigid Multi-Modal 3D Medical Image Registration Based on Foveated Modality Independent Neighborhood Descriptor

**DOI:** 10.3390/s19214675

**Published:** 2019-10-28

**Authors:** Feng Yang, Mingyue Ding, Xuming Zhang

**Affiliations:** 1 Department of Biomedical Engineering, School of Life Science and Technology, Ministry of Education Key Laboratory of Molecular Biophysics, Huazhong University of Science and Technology, Wuhan 430074, China; fyang@foxmail.com (F.Y.); myding@hust.edu.cn (M.D.); 2 School of Computer and Electronics and Information, Guangxi University, Nanning 530004, China

**Keywords:** medical image registration, similarity measure, non-rigid transformation, computational efficiency, registration accuracy

## Abstract

The non-rigid multi-modal three-dimensional (3D) medical image registration is highly challenging due to the difficulty in the construction of similarity measure and the solution of non-rigid transformation parameters. A novel structural representation based registration method is proposed to address these problems. Firstly, an improved modality independent neighborhood descriptor (MIND) that is based on the foveated nonlocal self-similarity is designed for the effective structural representations of 3D medical images to transform multi-modal image registration into mono-modal one. The sum of absolute differences between structural representations is computed as the similarity measure. Subsequently, the foveated MIND based spatial constraint is introduced into the Markov random field (MRF) optimization to reduce the number of transformation parameters and restrict the calculation of the energy function in the image region involving non-rigid deformation. Finally, the accurate and efficient 3D medical image registration is realized by minimizing the similarity measure based MRF energy function. Extensive experiments on 3D positron emission tomography (PET), computed tomography (CT), T1, T2, and (proton density) PD weighted magnetic resonance (MR) images with synthetic deformation demonstrate that the proposed method has higher computational efficiency and registration accuracy in terms of target registration error (TRE) than the registration methods that are based on the hybrid L-BFGS-B and cat swarm optimization (HLCSO), the sum of squared differences on entropy images, the MIND, and the self-similarity context (SSC) descriptor, except that it provides slightly bigger TRE than the HLCSO for CT-PET image registration. Experiments on real MR and ultrasound images with unknown deformation have also be done to demonstrate the practicality and superiority of the proposed method.

## 1. Introduction

In recent years, the non-rigid three-dimensional (3D) multi-modal medical image registration has attached significant attention [1,2,3,4]. This mainly stems from two aspects. Firstly, the different 3D imaging modalities are often fused to produce the precise diagnosis, since they can provide complementary information for interpreting the anatomy, tissue, and organ. As the necessary prerequisite for image fusion, multi-modal medical image registration is significant in relating clinically significant information from the different images. However, the relationship of intensity values in multi-modal 3D medical images might be highly complicated due to differences between the imaging principles, which leads to the difficulty in the construction of the appropriate similarity measure. Secondly, non-rigid deformation generally cannot be ignored for the soft organs that are easy to deform. Accordingly, the non-rigid transformation must be used as a deformation model in the non-rigid multi-modal medical image registration. However, the non-rigid transformation often involves numerous parameters, which will render accurate image registration difficult [5,6,7,8]. Therefore, the non-rigid multi-modal 3D medical image registration has become a challenging task [9,10,11,12].

The grey information and spatial information of 3D images are generally considered at the same time in order to construct a suitable similarity measure for non-rigid multi-modal 3D medical image registration. The typical similarity measure construction method is to combine the mutual information (MI) and spatial information [13]. Rueckert et al. [14] proposed using the second-order MI to encode the local information by considering both intensity information and structural information of images. However, this method needs to use a four-dimensional (4D) histogram to calculate the similarity measure. The number of grey levels cannot be too large in order to avoid the curse of dimensionality of high dimensional histograms. Pluim et al. [15] put forward a method that combines the normalized MI with the gradient amplitude and direction for rigid multi-modal image registration. Loeckx et al. [16] presented the image registration method that is based on the conditional MI. This method adopted the 3D joint histogram including the grey levels and the spatial information distribution of the reference and float images. However, the MI for 3D images in itself is computationally complicated and its combination with the spatial information will further lead to high computational complexity for the above registration methods.

The structural representation methods have been presented to more effectively measure the similarity between the different images [17,18,19,20,21,22]. Wachinger et al. [17] presented the entropy images based structural representation method. In this method, the entropy images were produced by calculating the histogram of image blocks, and then the sum of squared differences (SSD) on the entropy images was used as the similarity measure for image registration. As this method tends to produce the blurred entropy images, it cannot ensure the satisfactory registration results. Heinrich et al. [18] proposed a modality independent neighborhood descriptor (MIND) for the non-rigid multi-modal image registration. Based on the concept of image self-similarity that was introduced in non-local means image denoising, the MIND first extracted the distinctive and multi- dimensional features based on the intensity differences within a search region around each voxel in each modality. Subsequently, the SSD between MIND representations of two images was used as the similarity metric within a standard non-rigid registration framework. Although the MIND is robust to non-functional intensity relations and image noise, it cannot provide the effective structural representation for the complicated medical images with the weak, discontinuous, and complex details, because it only utilizes the similarity of image intensities. The self-similarity context (SSC) descriptor, an improved version of MIND, was proposed in [19]. The SSC descriptor was designed to find the context around the voxel of interest. The point-wise distance between SSC descriptors was used as the metric for the deformable registration on a minimum spanning tree while using dense displacement sampling (deeds) [20]. Zhu et al. [21] explored the self-similarity inspired local descriptor for structural representation based on the low-order Zernike moments with good robustness to image noise. This method cannot work well for ultrasound (US) images and positron emission tomography (PET) images with blurred features due to the ignorance of high-order Zernike moments with better feature representation ability than the low-order ones.

The solution of deformation parameters involved in the transformation model, as a high dimensional optimization problem, is a very challenging task apart from the difficulty in the construction of similarity measure for the non-rigid multi-modal 3D medical image registration. One approach to solve this problem is to use the local optimization methods (e.g., the L-BFGS-B method [23]), the global optimization methods (e.g., the evolutionary strategies [24] and the particle swarm optimization (PSO) [25]), as well as the combined methods (e.g., the hybrid L-BFGS-B and cat swarm optimization (HLCSO) method [26]). However, these methods cannot produce the satisfactory registration results in the case of the high-dimensional optimization problem. Another popular method is to reduce the dimension of transformation parameters while using the geometric transform models that are based on knowledge [27,28,29,30]. In these methods, it is required to have enough understanding of material properties of organs or tissues to establish a suitable geometric transform. However, some organs and tissues are so complicated that the existing methods cannot accurately characterize their material properties. Meanwhile, when determining the geometry and the boundary conditions, it is necessary to accurately segment the anatomy of medical images, which indeed is a very challenging task. Some alternative methods can be adopted to address this challenging problem. For example, by means of the mask image, the areas in the images that involve no non-rigid deformation can be covered up to reduce the number of the deformation field variables that are involved in the optimization process. However, the shape of such areas might often be irregular, thereby accurately leading to the difficulty in determining the mask image.

We have proposed a novel registration method using an improved modality independent neighborhood descriptor (MIND) based on the foveated nonlocal self-similarity to address these problems in the construction of similarity measure and the solution of non-rigid transformation parameters. The contributions of our work lie in the two aspects. For one thing, we have designed the foveated MIND (FMIND) for the effective structural representations of 3D medical images, thereby ensuring accurate image registration. One the other hand, the spatial constraint method based on the FMIND is proposed and introduced into the Markov random field (MRF) optimization to reduce the number of non-rigid transformation parameters and restrict the calculation of the energy function in the image regions involving local non-rigid deformation, thereby ensuring efficient image registration. Extensive experiments on multi-modal medical images demonstrate that the proposed method is provided with higher registration accuracy, except for computed tomography-positron emission tomography (CT-PET) images and higher computational efficiency than other evaluated registration methods.

## 2. Methods

### 2.1. The Framework of the FMIND Based Image Registration Method

**Figure 1:**
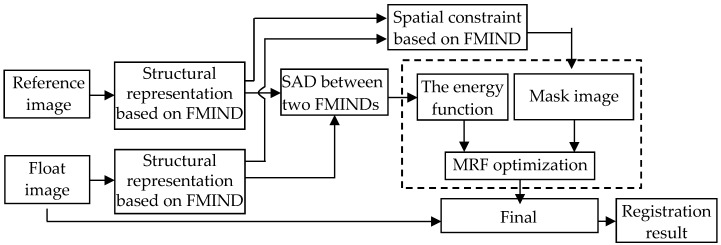
Flowchart of the foveated modality independent neighborhood descriptor (FMIND) based image registration.

Figure 1 shows the flowchart of the proposed image registration based on the FMIND. Firstly, the FMIND is constructed based on the foveated nonlocal self-similarity and it is applied to the reference image *I_R_* and the float image *I_F_* to produce the corresponding structural representations FMIND (*I_R_*) and FMIND (*I_F_*), respectively. Afterwards, the objective function, i.e., the energy function, is established based on the free-from deformation (FFD) model and the similarity measure defined as the sum of absolute differences (SAD) between FMIND(*I_R_*) and FMIND(*I_F_*). Finally, the FMIND based spatial constraint is introduced to produce the mask image for the MRF discrete optimization. During the iterative optimization, the deformation vector, which is a vector of parameters defining the deformation field, is produced at each iteration. The final optimal deformation vector *T*’ will be obtained once the optimization procedure is terminated, and it is utilized to produce the registration result.

### 2.2. The Foveated Modality Independent Neighborhood Descriptor

The FMIND is presented based on the characteristics of human visual system (HVS). In the HVS, the distribution of cone cells is uneven. The foveation has the highest density. If the foveation is taken as the center, the cell density will fall fast when it is extended around. The optic nerve cells have similar characteristics. Therefore, when we watch a point in an image, this point will have the highest sensitivity and the sensitivity will drop with the increasing distance to the point. Inspired by the characteristics of the HVS, Alessandro Foi et al. [31] have proposed calculating the patch similarity based on the Euclidean distance $d^{\;\mathrm{FOV}}$ between the the foveated patches, defined as:

(1) $$d^{\;\mathrm{FOV}}\left(I,\;x_{1},\;x_{2}\right)\;=\;\|I_{x_{1}}^{\;\mathrm{FOV}}-I_{x_{2}}^{\;\mathrm{FOV}}{\|}_{2}^{2}$$

where $I_{x_{1}}^{\;\mathrm{FOV}}$ and $I_{x_{2}}^{\;\mathrm{FOV}}$ denote the foveated patches that were obtained by foveating the image I at the two fixation points $x_{1}$ and $x_{2}$. By applying the foveation operator F to the image *I*, the foveated patch $I_{x}^{\;\mathrm{FOV}}$ is produced as:

(2) $$I_{x}^{\;\mathrm{FOV}}\left(u\right)\;=\;F\left[I,\;x\right]\left(u\right),\;u\in S$$

where u denotes the location of any pixel in the foveated image patch S. In [31], the designed foveation operators mainly include the isotropic and anisotropic foveation operators. As the latter has more advantages than the former in describing the image edges and textures, it will be used as the foveation operator. This operator is defined as:

(3) $$F_{\rho ,\theta }\left[I,\;x\right]\left(u\right)\;=\;\underset{\xi \in Z^{2}}{\sum }I\left(\xi +x\right)v_{u}^{\rho ,\theta }\left(\xi -u\right),\;\forall u\in S$$

where $v_{u}^{\rho ,\theta }$ denotes the blur kernel and it is mainly structured by the elliptical Gaussian probability density function (PDF), ρ determines the elongation of the Gaussian PDF, and θ denotes the angular offset, respectively. The blur kernel $v_{u}^{\rho ,\theta }$ is defined as [31]:

(4) $$v_{u}^{\rho ,\theta }=\{\begin{array}{c} \sqrt{K\left(0\right)}g_{\frac{1}{2\sqrt{\pi }}\sqrt{\frac{K\left(0\right)}{K\left(u\right)}}}^{\rho ,∠u+\theta }\;u\;\neq \;0\\ \sqrt{K\left(0\right)}g_{\frac{1}{2\sqrt{\pi }}}\;u\;=\;0 \end{array}$$

where $\sqrt{K\left(0\right)}=\|v_{u}{\|}_{1}$, $\sqrt{K\left(u\right)}=\|v_{u}{\|}_{2}$, $g_{\frac{1}{2\sqrt{\pi }}}$ denote the elliptical Gaussian PDF with the standard deviation of $\frac{1}{2\sqrt{\pi }}$ and ∠*u*+θ determines the orientation of the axes of the elliptical Gaussian PDF.

**Figure 2:**
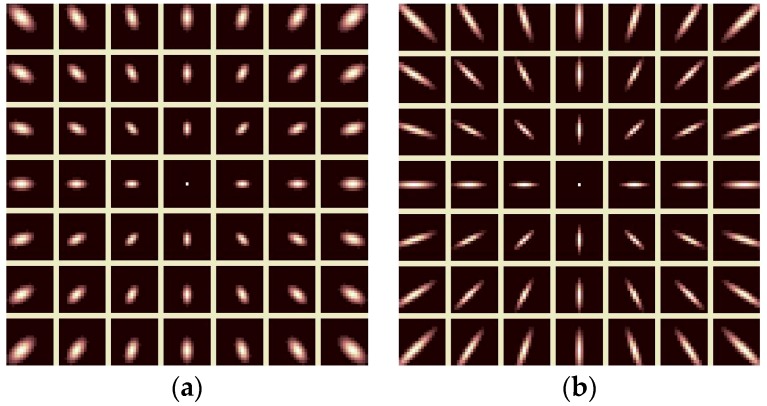
Anisotropic foveation operators with a 7 × 7 foveated patch and θ = 0. (**a**) ρ = 2; and, (**b**) ρ = 6.

Figure 2 gives an example of two anisotropic foveation operators, where S is a 7 × 7 foveated patch, θ = 0, and the different kernel elongation parameters ρ = 2 and ρ = 6 are used, respectively. Clearly, this radial design of these anisotropic foveation operators is consistent with HVS features, which thereby leads to the effective structural representation of images for the FMIND.

We will propose the FMIND based on the foveated nonlocal self-similarity between different image patches in the same image borrowing the idea of self-similarity in the non-local means denoising. The FMIND is expressed as:

(5) $$\mathrm{FMIND}\left(I,\;x,\;r\right)\;=\;\frac{1}{n}\exp\left(-\frac{d^{\;\mathrm{FOV}}\left(I,\;x,\;x+r\right)}{V^{\;\mathrm{FOV}}\left(I,\;x\right)}\right)\;r\in R$$

where R denotes a search window centered at *x*, $d^{\;\mathrm{FOV}}\left(I,\;x,\;x+r\right)$ denotes the distance between the foveated image patches $I_{x}^{\;\mathrm{FOV}}$ and $I_{x+r}^{\;\mathrm{FOV}}$; *n* is a normalization constant to ensure that the maximum of FMIND(I, x, r) is 1; $V^{\;\mathrm{FOV}}\left(I,\;x\right)$ denotes the variance of the foveated image patch $I_{x}^{\;\mathrm{FOV}}$ centered at *x* in the image *I*, and it controls the attenuation degree of this function in Equation (5). The variance $V^{\;\mathrm{FOV}}\left(I,\;x\right)$ is estimated as the mean of foveated distances for all the pixels in the foveated patch S.

(6) $$V^{\;\mathrm{FOV}}\left(I,\;x\right)=\frac{1}{\left|S\right|}\underset{m\in S}{\sum }d^{\;\mathrm{FOV}}\left(I,\;x,\;x+m\right)$$

where |S| denotes the number of pixels in S.

The structural information around the pixel *x* in the image *I* will be described by one-dimensional vector of size |R|, where |*R*| denotes the number of pixels in the search window *R* by means of the FMIND. After obtaining the FMIND for the reference and float images, the similarity metric SADF(I(x), J(x)) between two pixels at the same position *x* in the images *I* and *J* can be expressed as the mean SAD between FMIND(I, x, r) and FMIND(J, x, r) of pixels in *R*.

(7) $$\mathrm{SADF}\left(I\left(x\right),\;J\left(x\right)\right)=\frac{1}{\left|R\right|}\underset{r\in R}{\sum }\left|\mathrm{FMIND}\left(I,\;x,\;r\right)-\mathrm{FMIND}\left(J,\;x,\;r\right)\right|$$

where *R* takes a six-neighborhood in this paper.

### 2.3. MRF Optimization Based on the Spatial Constraint

#### 2.3.1. Discrete Optimization Based on the MRF

After obtaining the similarity measure for the two different modal images, we will use the FFD as the transformation model and use Markov random field (MRF) optimization [32] to obtain the transformation parameters in the FFD. The reason for choosing this discrete optimization method is that it does not need to calculate the gradient of the energy function in the process of optimization, which thereby facilitates producing the good registration result by avoiding falling into the local minimum. In this method, the image registration problem will be converted into the MRF based discrete optimization problem.

(8) $$E_{\mathrm{MRF}}\left(l\right)=\frac{1}{\left|G\right|}\underset{p\in G}{\sum }\left(V_{p}\left(l_{p}\right)+\lambda \underset{q\in N(p)}{\sum }V_{pq}\left(l_{p},\;l_{q}\right)\right)$$

(9) $$V_{p}\left(l_{p}\right)=\int_{\Omega }\mathrm{SADF}\left(I\left(x\right),\;J\circ T_{l_{p}}\left(x\right)\right)dx$$

(10) $$V_{pq}\left(l_{p},\;l_{q}\right)=\|T_{l_{p}}-T_{l_{q}}{\|}_{1}$$

where *E* denotes the general form of a first-order MRF, i.e., the energy function and λ is a constant; *G* is the set of vertices and |G| denotes the number of vertices in *G*, where *G* can be regarded as the vertex set in the FFD, because this method uses the FFD as the deformation model; N(p) and N(q) refer to the neighborhood of vertices *p* and *q*, respectively; l is the discrete labelling while $l_{p}$ and $l_{q}$ are the labels that are assigned to the vertices *p* and *q*, respectively; $V_{p}\left(l_{p}\right)$ denotes the data item of the energy function $E_{\mathrm{MRF}}\left(l\right)$, while $V_{pq}\left(l_{p},\;l_{q}\right)$ represents its smooth regularization and it takes the *L*_1_-norm to encourage the neighboring nodes *p* and *q* to keep the displacement.

Accordingly, the MRF optimization, actually, is to seek to assign a label that is associated with the deformation to each vertex, so that the energy function in Equation (8) is minimized. In this paper, the fast primal-dual (Fast-PD) [33] algorithm will be used for the MRF optimization to produce the registration result. More details about the Fast-PD algorithm can be found in [33].

#### 2.3.2. Spatial Constraint Based on the FMIND

When the above registration method is applied to three-dimensional (3D) medical images, the number of deformation field variables will be large. If all pixels’ displacement along the *x*, *y*, and *z* directions is considered, the number of dense deformation field variables will be 3$\cdot \left|I_{x}\right|\cdot$|$I_{y}$|·|$I_{z}$|, where $\left|I_{x}\right|,$ |$I_{y}$| and |$I_{z}$| denote the number of pixels in the *x*, *y,* and *z* dimensions of the image *I*, respectively. For example, there will be 50,331,648 deformation field variables when $\left|I_{x}\right|=$|$I_{y}$|=|$I_{z}$| = 256. It is indeed very time-consuming to address such a high dimensional optimization problem.

In the reference and float images, sometimes only some areas involve non-rigid deformation. In addition, the non-rigid registration is unnecessary for some smooth areas. For these regions, the mask can be used to indicate that they will be excluded from the registration process. In this way, we can not only reduce the number of variables for describing the deformation field, but also focus the calculation of the energy function in the image areas that indeed involve the local non-rigid deformation. However, the shape of areas without non-rigid deformation is often irregular. Generally, manual intervention or image segmentation is needed for obtaining the appropriate mask image. However, these technologies cannot ensure that the satisfactory mask image can be produced for US and PET images due to their low image contrast, blurriness, and edge discontinuousness.

We will put forward the spatial constraint method based on the FMIND to address the above problem. From Equation (7), it can be seen that SADF(I(x), J(x)) contains the corresponding relationship of the local spatial information at the pixel *x* in the images *I* and *J*. This information can be used to reduce the number of variables for describing the deformation field and limit the control nodes in the deformation field to move in the areas with the local non-rigid deformation. In the FMIND based spatial constraint method, the vertex set *G* will be divided into the set $G_{s}$ of static vertices and the set $G_{d}$ of dynamic vertices based on the local spatial information included in the FMIND. The vertices in $G_{s}$ are similar to those in the smooth areas and the areas that involve no deformation. Meanwhile, the vertices in $G_{d}$ are similar to those in the non-smooth areas involving the deformation. The calculation of the energy function can be restricted in the areas involving the non-rigid deformation through the movement of these dynamic vertices with the local non-rigid deformation. In this way, the number of deformation field variables will decrease from 3$\cdot \left|G_{x}\right|\cdot$|$G_{y}$|·|$G_{z}$| to 3·|$G_{d,x}$|·|$G_{d,y}$|·|$G_{d,z}$|, where |$G_{d,x}$|, |$G_{d,y}$|, and |$G_{d,z}$| denote the number of vertices in *x*, *y*, and *z* dimensions of $G_{d}$. By utilizing the FMIND based spatial constraint, we can obtain the division of vertices, thereby generating the mask image without manual intervention and image segmentation.

**Figure 3:**
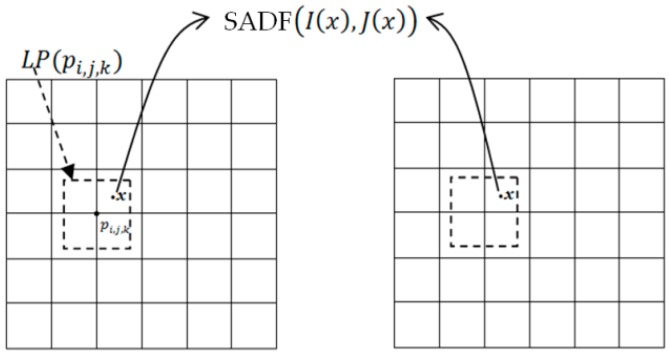
The illustration of the local image block $LP(p_{i,\;j,\;k}).$

There will be two requirements that no vertices of the whole MRF model will be omitted and no repeated division of vertices will be done to ensure the effective division of *G* in the FMIND based spatial constraint method. Correspondingly, the logical relationship among *G*, $G_{s}$, and $G_{d}$ can be expressed as $G\;=\;G_{s}\cup G_{d}$ and $\emptyset \;=\;G_{s}\;\cap \;G_{d}$, where ∅ denotes the empty set. We have designed the following vertex partition algorithm according to the above requirements. For any vertex $p_{i,\;j,\;k}$ of the set *G* in the MRF model, i.e., $G\;=\;\left\{p_{i,\;j,\;k}|1\leq \;i\;\leq {|G}_{x}|,\;1\leq \;j\;\leq {|G}_{y}|,\;1\leq \;k\;\leq {|G}_{z}|\right\},\;$we will check the similarity metric SADF for each pixel *x* in the local image patch $LP(p_{i,\;j,\;k})$ with radius $R_{LP}$, which takes $p_{i,\;j,\;k}$ in *G* as the center, as shown in Figure 3. Let *con* denote the number of pixels in $LP(p_{i,\;j,\;k})$ whose similarity 1-SADF(I(x), J(x)) is greater than a certain threshold δ. If the ratio of *con* to the patch size $\left|LP(p_{i,\;j,\;k})\right|$ is greater than the static factor ε, $p_{i,\;j,\;k}$ will be regarded as a static vertex. In a similar way, we can determine other static vertices to generate the final set $G_{s}$. Accordingly, $G_{d}$ will be easily computed as $G_{d}=G\;-\;G_{s}$.

Obviously, the performance of the vertex partition algorithm depends on three key parameters $R_{LP}$, δ, and ε. Here, δ will influence the decision of whether two pixels are similar and ε is used to adjust the probability that $p_{i,\;j,\;k}$ is divided into $G_{s}$. Section 3.1 discusses the choice of these parameters. Algorithm 1 shows the detailed implementation of the proposed vertex partition algorithm.

**Algorithm 1.** Partition of the vertex set *G***Input**: FMIND(I, x, r), FMIND(J, x, r), G, δ, ε, $R_{LP}$**Output**: $G_{s},\;G_{d}$(1) $G_{s}=\emptyset$;(2) ***for*** (*i* = 1; *i* ≤ |$G_{x}$|; *i* = *i*++)(3) ***for*** (*j* = 1; *j* ≤ |$G_{y}$|; *j* = *j*++)(4) ***for*** (*k* = 1; *k* ≤ |$G_{z}$|; *k* = *k*++)(5) *con* = 0;(6) ***while***
$x\in LP(p_{i,\;j,\;k}$)(7) ***if*** (1-SADF(I(x), J(x))>δ)(8) *con*++;(9) ***end if***(10) ***end while***(11) ***if***
$\left(\frac{con}{\left|LP(p_{i,\;j,\;k})\right|}>\varepsilon \right)$
(12) $G_{s}\;=\;p_{i,\;j,\;k}\cup G_{s}$;(13) ***end if***(14) ***end for***(15) ***end for***(16) ***end for***(17) $G_{d}\;$ = $G\;-\;G_{s}$;(18) ***return***
$G_{s},\;G_{d}$;

## 3. Results

In this section, we will first discuss the selection of several key parameters in the FMIND method, and then use the method based on the anatomical landmarks selected by doctors to compare registration accuracy and efficiency of the proposed FMIND method with those of the entropy images based SSD (ESSD) [17], MIND [18], SSC [19], and HLCSO [26] methods. For the appreciation of registration performance, we have used four datasets with synthetic deformation, including simulated 3D MR images in BrainWeb database [34], 3D CT and MR images in NA-MIC database [35], 3D CT and PET images in NA-MIC database [36], and real 3D MR images from Retrospective Image Registration Evaluation project [37]. Besides, we have used the real MR and US images with unknown real deformation in the Brain Images of Tumors for Evaluation (BITE) database [38] available at [39] to appreciate the practicality of the proposed method. Here, the implementation efficiency of the evaluated registration methods is appreciated by their running time. For all evaluated methods, they are implemented on the personal computer with 2.40 GHz CPU and 4 GB RAM while using the mixed programming of Matlab and C++.

**Figure 4:**
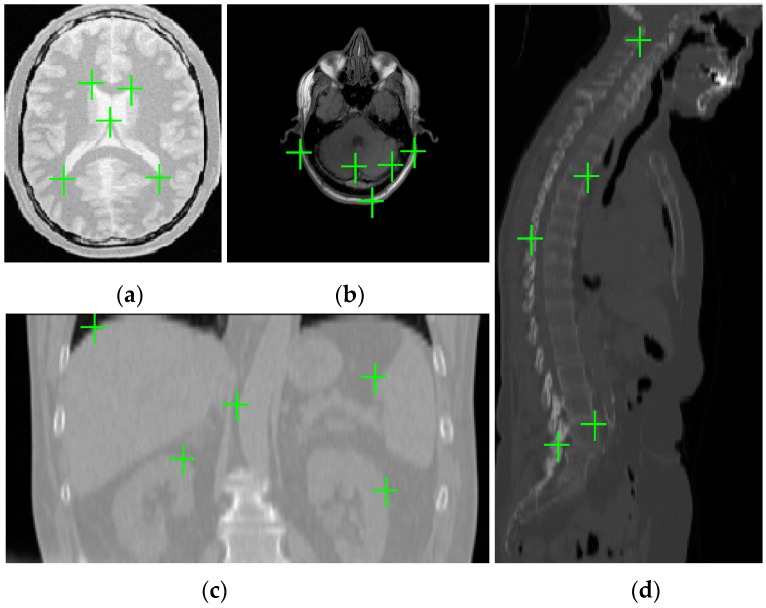
Landmarks in one slice of three-dimensional (3D) medical images. (**a**) simulated proton density (PD) weighted image; (**b**) real T1 weighted image; (**c**) abdomen computed tomography (CT) image; and, (**d**) whole-body CT image.

In the case of synthetic deformation, the registration accuracy is appreciated by the target registration error (TRE) [40], defined as:

(11) $$\mathrm{TRE}\;=\;\frac{1}{N}\sum_{i=1}^{N}\sqrt{{\left(T_{L_{x}}-T_{D_{x}}\right)}^{2}+{\left(T_{L_{y}}{-T}_{D_{y}}\right)}^{2}+{\left(T_{L_{z}}{-T}_{D_{z}}\right)}^{2}}$$

where $T_{L}$ is the synthetic deformation (i.e., the ground truth generated by using a linear combination of radial basis functions), $T_{D}$ is the deformation that is estimated by the registration methods, and *N* denotes the number of landmarks selected manually based on doctors’ advice from the reference images. For each pair of reference and float images, different synthetic deformations will be applied to the float image for 25 times and we will manually select 90 (*N* = 90) landmarks from each 3D reference image to compute the TRE. The mean of TREs values for registering 25 deformed images will be used to appreciate the registration accuracy. Figure 4 gives an example of chosen landmarks in one slice of simulated 3D PD weighted image and real 3D T1 weighted image for MR image registration, 3D CT image for CT-MR image registration and 3D CT image for CT-PET image registration.

### 3.1. Parameter Setting

In the FMIND method, we will fix θ = 0, S to be a 5 × 5 foveated patch in Equation (3) and λ = 0.01 in Equation (8). The remaining parameters include the kernel elongation parameter ρ, the image patch radius *R_LP_*, the similarity threshold δ, and the static factor ε. We will conduct experiments on three pairs of simulated MR images (T2-T1, PD-T2, and PD-T1) from BrainWeb database in order to effectively determine these parameters, where the former and the latter will be used as the reference and float images, respectively. These simulated MR images are realistic MRI data volumes that are produced by an MRI simulator while using three sequences (T1, T2, and PD weighted) and a variety of slice thicknesses, noise levels, and levels of intensity non-uniformity.

#### 3.1.1. The Kernel Elongation Parameter ρ

**Figure 5:**
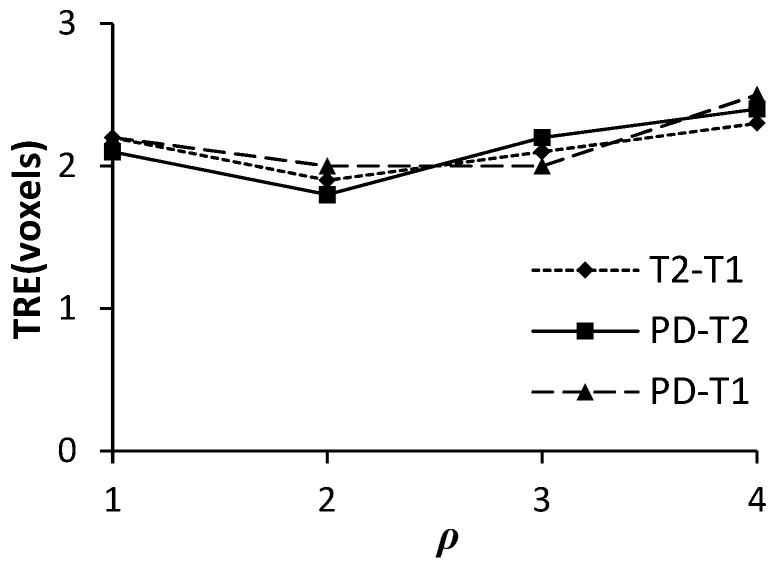
The target registration error (TRE) with different ρ values.

Figure 5 shows the TRE values of the FMIND method using different ρ values. For the purpose of evaluating the influence of ρ on registration accuracy, we have set the significant level α = 0.05 in one-way Analysis of Variance (ANOVA) [41]. The obtained significance value *P* is 0.001, which means that ρ has a significant impact on registration accuracy of the FMIND method. From Figure 5, we can see that the TRE achieves the minimum value when ρ = 2. Thus, we have fixed ρ = 2 in the proposed method.

#### 3.1.2. The Image Patch Radius *R_LP_*

**Figure 6:**
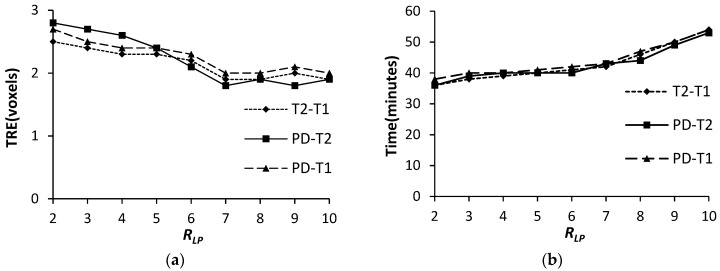
The TRE and computation time with different *R_LP_*. (**a**) TRE (voxels); (**b**) Time (minutes).

Figure 6 shows the TRE values and computational time of the FMIND method while using the different *R_LP_* values. Likewise, one-way ANOVA with *α* = 0.05 is used to evaluate the influence of *R_LP_* on the registration results. The obtained significance value *P* is 0.001 for registration accuracy and *P* is 0.007 for registration efficiency. Therefore, *R_LP_* has the significant impact on both registration accuracy and efficiency. It can be seen from Figure 6a that the TRE significantly declines when *R_LP_* varies from 2 to 7, and it tends to be stable when *R_LP_* varies from 7 to 10. Besides, Figure 6b indicates that the registration time gradually increases for the increasing *R_LP_*. The reason is that, for a larger *R_LP_*, the more pixels need to be processed in the vertex partition algorithm. Therefore, we have set *R_LP_* as 7 to achieve the trade-off between registration accuracy and efficiency.

#### 3.1.3. The Similarity Threshold δ

**Figure 7:**
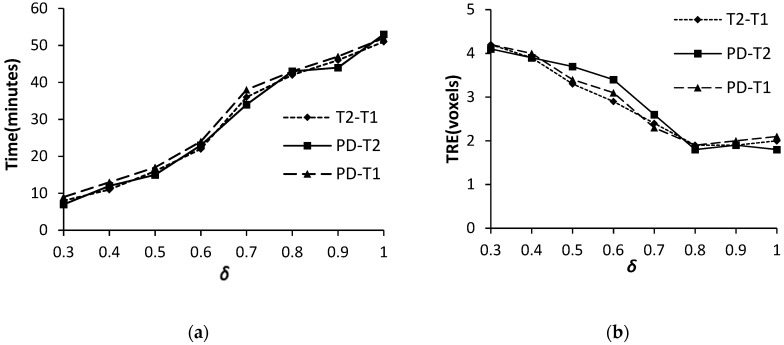
The TRE and computation time with different δ. (**a**) TRE (voxels); and, (**b**) Time (minutes).

Figure 7 shows the effect of δ. For one-way ANOVA with α = 0.05, the obtained *P* values are 0.001 for both registration accuracy and efficiency, which means the significant impact of δ on the registration performance of the FMIND method. From Figure 7a, we can see that the TRE significantly declines when δ varies from 0.3 to 0.8. The reason is that for a larger δ in this range, fewer control vertices are divided into the static ones and the number of dynamic control vertices increases, thereby resulting in a smaller TRE. Meanwhile, the TRE tends to be stable when δ varies from 0.8 to 1.0. The reason is that, for the threshold δ in this range, the number of dynamic control vertices will increase to a certain value, so that the variation of δ will have little effect on the TRE. Besides, Figure 7b indicates that the registration time significantly increases with the increasing δ. It is easy to understand that, for a larger δ, the increasing dynamic control vertices will lead to more processing time. Therefore, we set δ as 0.8 to balance the registration accuracy and efficiency.

#### 3.1.4. The Static Factor ε

**Figure 8:**
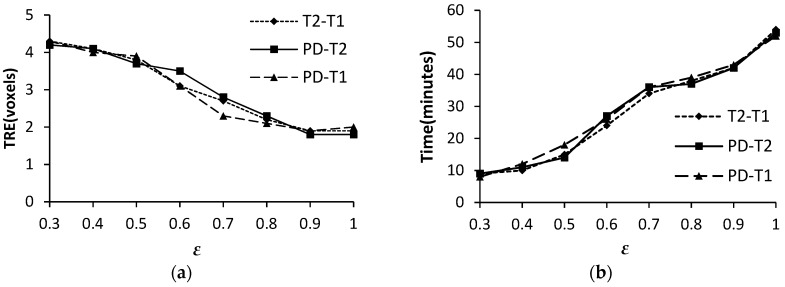
The TRE and computation time with different ε. (**a**) TRE (voxels); and, (**b**) Time (minutes).

Figure 8 shows the effect of the static factor ε on registration accuracy and efficiency. As ε is also used to divide the control vertices, it has a similar effect on registration performance to the similarity threshold δ. According to Figure 8a,b, ε has the opposite effect on TRE and computational time. We have chosen ε = 0.9 for the FMIND method based on the comprehensive consideration of registration performance.

### 3.2. Comparison of Registration Performance

#### 3.2.1. Registration Results of Simulated T1, T2 and PD Images

In order to quantitatively and qualitatively compare the registration performance of the FMIND method and other methods on 3D T1, T2 and PD weighted MR images, we will test them on three pairs of simulated T2-T1, PD-T2, and PD-T1 images of size 256 × 256 × 32. For all evaluated methods, the mean and the standard deviation (std) of TRE values as well as the *P* values for the *t*-test with the significance level *α* = 0.05 are computed and are shown in Table 1. In Table 1, “/” means that no registration is implemented. It is shown that all of the *P* values are less than 0.002, which indicates that there exists significant difference between the FMIND method and any other compared method in terms of TRE. Specifically, as regards the registration of T2-T1 images, the mean and the standard deviation of TRE values for the MIND method are 2.2 voxel and 0.5 voxel, respectively. By comparison, the FMIND method has the lower mean (1.8 voxel) and standard deviation (0.2 voxel) of TRE values than the MIND method. This is mainly due to the advantage of the proposed FMIND in describing the structural information of multi-modal MR images over the MIND method.

**Figure 9:**
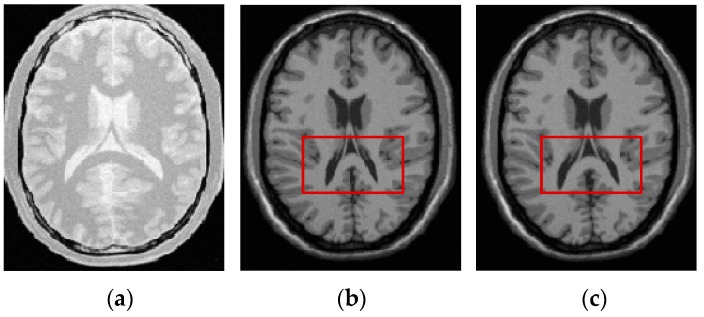

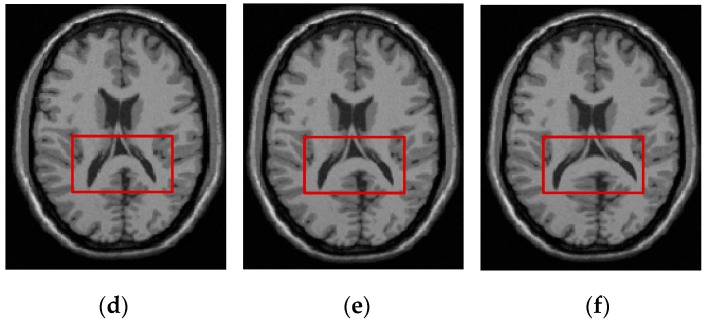
The registration results of all evaluated methods operating on 3D PD-T1 images. (**a**) PD image (reference image); (**b**) T1 image (float image); (**c**) ESSD; (**d**) MIND; (**e**) hybrid L-BFGS-B and cat swarm optimization (HLCSO); and, (**f**) FMIND.

Figure 9 visually shows the registration results of 3D PD-T1 images for all the evaluated methods. Here, it should be noted that the background regions in these images are removed and the same operation will be implemented for other experiments in the rest of this paper. The comparison among Figure 9f and Figure 9c–e shows that the registration result of the FMIND method is more similar to the reference image that is shown in Figure 9a than those of the ESSD, MIND, and HLCSO methods. Especially for the tissue indicated by the red boxes in Figure 9, the FMIND can recover its deformation better than other evaluated methods.

Table 2 lists the implementation time of all evaluated methods on 3D T1, T2, and PD weighted MR images. It can be observed that the FMIND method, on average, takes approximately 44 min to produce the registration results, and it has the highest computational efficiency among all of the methods. The reason lies in that the FMIND method can generally reduce the number of deformation field variables by utilizing the FMIND based spatial constraint for MRF optimization.

#### 3.2.2. Registration Results of CT and MR Images

To appreciate registration performance of all evaluated methods operating on CT and MR images from NA-MIC database, these methods are implemented to correct the synthetic deformation that is applied to the float image, where the CT and MR images will be used as the reference and float images, respectively. Here, the liver CT and MR images of size 256 × 256 × 32 are intra-operatively and pre-operatively acquired, respectively. Due to strong differences in image contrast between CT and MR images, their registration is difficult.

Table 3 lists the TRE and *P* values of *t*-test for the FMIND method and other methods. As you can see, among all the compared methods, the FMIND method has the highest registration accuracy by providing the lower TRE than other methods. Meanwhile, all the *P* values are less than 0.003, which indicates the significant difference between the FMIND method and any other method in terms of TRE.

**Figure 10:**
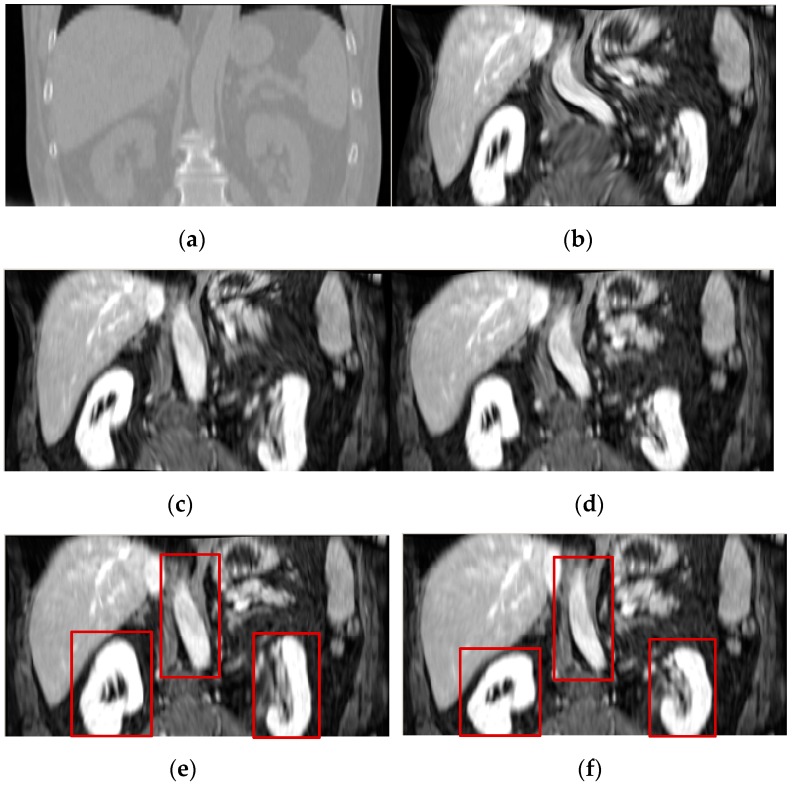
The registration results of all evaluated methods operating on 3D CT–MR images. (**a**) CT image (reference image); (**b**) MR image (float image); (**c**) ESSD; (**d**) MIND; (**e**) HLCSO; and, (**f**) FMIND.

Figure 10 shows the registration results of 3D CT-MR images for all the evaluated methods. As shown in Figure 10c,d, the ESSD and MIND method cannot effectively correct the deformation that is involved in the MR image. The FMIND method can produce a more similar registration result to the reference image that is shown in Figure 10a than the ESSD and MIND methods. When compared with the most competitive HLCSO method, the proposed method performs better in that it can correct the deformation of some tissues more effectively, as indicated by the three red boxes that are shown in Figure 10e,f.

Table 4 lists the implementation time of all the evaluated methods. The comparison indicates the advantage of the FMIND method in computational efficiency. Here, it should be noted that the implementation time for all evaluated registration methods in Table 4 is very similar to that in Table 2, because the used CT and MR images have the same size (256 × 256 × 32) to T1, T2, and PD images.

#### 3.2.3. Registration Results of CT and PET Images

The 3D whole body CT-PET images from NA-MIC database are also used to demonstrate the advantage of the FMIND method. Here, the CT and PET images of size 168 × 168 × 149 are the reference image and the float image, respectively. It is difficult to realize accurate registration of CT images and blurry PET images of low resolution.

**Figure 11:**
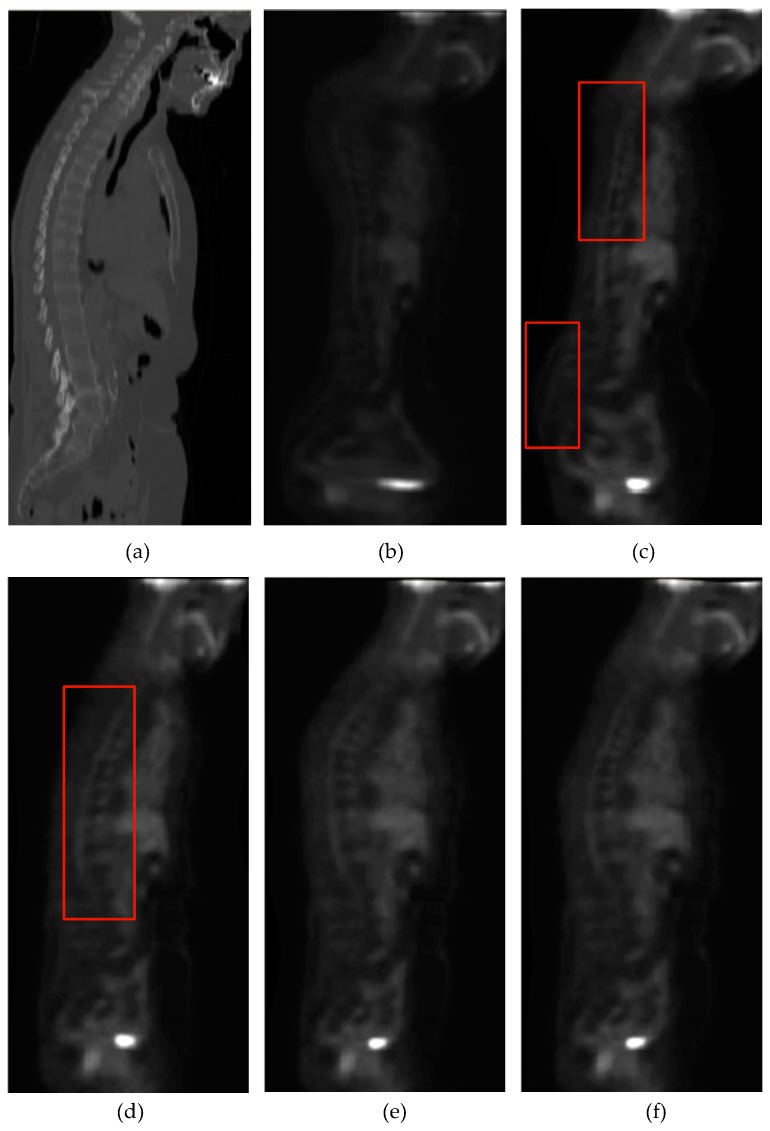
Registration results of all evaluated methods operating on the 3D CT–PET images. (**a**) CT (reference image); (**b**) PET (float image); (**c**) ESSD; (**d**) MIND; (**e**) HLCSO; and, (**f**) FMIND.

Figure 11 shows the registration results of the 3D CT-PET images for the ESSD, MIND and HLCSO, and FMIND methods. It can be observed that the ESSD and MIND methods cannot correct the deformation in the regions that are marked with the red boxes in Figure 11c,d well. By comparison, the registration results of the HLCSO and FMIND methods are more similar to the reference image shown in Figure 11a than the ESSD and MIND methods.

Table 5 lists the mean and standard deviation of TRE for all evaluated methods operating on 3D CT and PET images. The comparison of TRE values shows that the HLCSO method provides the minimum mean (2.6 voxel) and standard deviation (0.7 voxel) of TRE among all the compared methods. However, the mean (2.8 voxel) and standard deviation (0.9 voxel) of TRE for the FMIND method are lower than those for the ESSD and MIND methods. The reason can be explained in this way. For the PET image, its contrast and resolution are poor and the edge features are not obvious. Therefore, the FMIND method is slightly inferior to the HLCSO method in the registration of 3D CT-PET images. However, the proposed FMIND method can still provide better structural representation results than the ESSD and MIND methods, thereby leading to its improved registration accuracy than the latter.

Table 6 lists the calculation time of the various methods operating on CT and PET images. It can be seen from Table 6 that, as compared with other methods, the calculation time of the FMIND method is significantly reduced because the spatial constraint based on the FMIND, to a certain extent, helps to reduce the number of variables that are required by the deformation model. Especially, when compared with the HLCSO method, although the FMIND method has slightly lower registration accuracy, its computational efficiency is more than two times higher. Besides, as compared with the calculation time listed in Table 2, more calculation time will be involved in the registration of CT-PET images because their size (168 × 168 × 149) is bigger than that of T1, T2, and PD images.

#### 3.2.4. Registration Results of Real MR Images

The MR images from RIRE database are chosen to verify the superiority of the FMIND method in registering the real MR images, where the T1 and PD weighted MR images are used as the reference and float images, respectively. These MR images were acquired while using a Siemens SP Tesla scanner, among which the T1 and PD image volumes were obtained with an echo time of 15 ms and 20 ms, respectively [42].

Here, we will only compare the proposed method with the MIND and SSC methods, which are most similar to our method. Table 7 lists the TRE values of the three methods. Clearly, the SSC method generally provides slightly smaller TRE values than the MIND methods. The two methods are outperformed by the FMIND method in terms of registration accuracy. The comparison of TRE values indeed demonstrates the effectiveness and advantage of the FMIND method in correcting the deformation of real MR images.

#### 3.2.5. Registration Results of Real MR and US Images

We will use the pre-operative T1 weighted MR and intra-operative post-resection US images of 13 patients [43] from BITE database for registration performance appreciation to further demonstrate the practicality of the FMIND method. In [43], the MR images were obtained a few days before the surgery while the post-resection 2D US images were acquired while using Philips HDI 5000 ultrasound machine with a P7-4 MHz phased array transducer and they were reconstructed into ultrasound volume with a voxel size of 1 mm. The used MR data contain the tumor, which is replaced by the resection cavity, and thus will not exist in the post-resection US images. Therefore, to register 3D MR to 3D US images is highly challenging. For each patient, 15 landmarks in average selected in [43] are used for TRE evaluation.

Table 8 lists the TRE values of the MIND, SSC, and FMIND methods. Clearly, the SSC method provides smaller TRE values than the MIND method. The FMIND method also performs better than the MIND method, in that the introduction of foveated nonlocal self-similarity ensures more effective structural representations of MR and US images. Please note that the proposed method cannot significantly outperform the SSC method for registration of US-MR images due to the disadvantageous influence of speckle noise that is inherent in US images.

## 4. Conclusions

In this paper, we have proposed a novel non-rigid multi-modal 3D medical image registration method that is based on the foveated independent neighborhood descriptor. The advantages of the proposed method lie in two aspects. Firstly, the proposed FMIND can effectively capture the structural feature information of 3D medical images, thereby providing better structural representations than the existing approaches. Secondly, the FMIND based spatial constraint method can help to reduce the number of non-rigid transformation parameters because the FMIND contains the corresponding relationship of the local spatial information at the same pixel in the reference and float images, thereby providing an effective means for solving the high-dimensional optimization problem that is involved in the medical image registration. Experiments on 3D ultrasound, CT, PET, T1, T2, and PD weighted MR images demonstrate that our method can provide higher computational efficiency and higher registration accuracy as compared with the HLCSO, ESSD, MIND and SSC methods, except that its TRE is slightly bigger than that of the HLCSO for CT-PET image registration. Future work will be focused on the acceleration of the method without compromising registration accuracy by using sparse data sampling and parallel data processing strategies to facilitate its clinical applications.

## Figures and Tables

**Table 1:** The TRE for all evaluated methods and the *P* values for the *t*-test between the FMIND method and other compared methods operating on the T2-T1, PD-T2, and PD-T1 image pairs.

Methods	TRE (Voxels)								
	T2-T1			PD-T2			PD-T1		
	Mean	Std	*P*	Mean	Std	*P*	Mean	Std	*P*
**/**	4.8	2.7		4.8	2.7		4.9	2.9	
**ESSD**	2.7	0.8	2.8 × 10^−4^	2.8	0.8	4.4 × 10^−4^	2.9	0.9	8.2 × 10^−4^
**MIND**	2.2	0.5	6.4 × 10^−4^	2.3	0.6	5.2 × 10^−4^	2.3	0.5	3.2 × 10^−4^
**HLCSO**	2.0	0.2	1.2 × 10^−3^	2.1	0.3	1.7 × 10^−3^	2.2	0.4	2.6 × 10^−4^
**FMIND**	1.8	0.2		1.9	0.3		2.0	0.3	

**Table 2:** Computation time for all evaluated methods operating on the T2-T1, PD-T2, and PD-T1 image pairs.

Methods	Time (Minutes)					
	T2-T1		PD-T2		PD-T1	
	Mean	Std	Mean	Std	Mean	Std
**ESSD**	53.8	7.4	52.2	9.6	55.3	8.8
**MIND**	62.2	11.4	61.7	11.1	63.5	13.9
**HLCSO**	98.4	18.8	97.2	17.4	102.6	21.5
**FMIND**	44.2	7.2	45.4	6.8	46.3	8.0

**Table 3:** The TRE for all evaluated methods and the *P* values for the *t*-test between the FMIND method and other compared methods operating on the three-dimensional CT-magnetic resonance (3D CT–MR) image pairs.

Methods	TRE (Voxels)		
	Mean	Std	*P*
**/**	6.7	2.9	
**ESSD**	3.3	1.0	4.7 × 10^−4^
**MIND**	2.7	0.8	2.9 × 10^−3^
**HLCSO**	2.5	0.7	1.6 × 10^−3^
**FMIND**	2.3	0.7	

**Table 4:** Computation time for all evaluated methods operating on the 3D CT–MR image pairs.

Methods	Time (Minutes)	
	Mean	Std
**ESSD**	54.6	7.6
**MIND**	64.4	11.8
**HLCSO**	101.2	19.2
**FMIND**	45.0	7.3

**Table 5:** TRE for all evaluated methods operating on the 3D CT–PET image pairs.

Methods	TRE (Voxels)	
	Mean	Std
**/**	5.7	2.8
**ESSD**	3.6	1.6
**MIND**	3.1	1.2
**HLCSO**	2.6	0.7
**FMIND**	2.8	0.9

**Table 6:** Computation time for all evaluated methods operating on the 3D CT–PET image pairs.

Methods	Time (Minutes)	
	Mean	Std
**ESSD**	106.2	19.4
**MIND**	124.8	22.2
**HLCSO**	198.6	30.2
**FMIND**	88.2	13.2

**Table 7:** The TRE for the MIND, self-similarity context (SSC), and FMIND methods operating on the real T1-PD image pairs.

Methods	TRE (Voxels)	
	Mean	Std
**/**	4.0	1.8
**MIND**	2.4	0.4
**SSC**	2.3	0.4
**FMIND**	2.1	0.3

**Table 8:** The TRE for the MIND, SSC, and FMIND methods operating on the real US-MR images of 13 patients.

Methods	TRE (mm)	
	Mean	Std
**/**	5.9	3.2
**MIND**	3.6	1.0
**SSC**	3.3	0.9
**FMIND**	3.2	0.9

## References

[1] Maintz J.A., Viergever M.A. (1998). A survey of medical image registration. Med. Image Anal..

[2] Zitova B., Flusser J. (2003). Image registration methods: A survey. Image Vis. Comput..

[3] Sotiras A., Davatzikos C., Paragios N. (2013). Deformable medical image registration: A survey. IEEE Trans. Med. Imaging.

[4] Viergever M.A., Maintz J.B.A., Klein S., Murphy K., Staring M., Pluim J.P.W. (2016). A survey of medical image registration—under review. Med. Image Anal..

[5] Yang F., Ding M., Zhang X., Wu Y., Hu J. (2013). Two phase non-rigid multi-modal image registration using weber local descriptor-based similarity metrics and normalized mutual information. Sensors.

[6] Zhang Z., Han D., Dezert J., Yang Y. (2019). A new image registration algorithm based on evidential reasoning. Sensors.

[7] Ferreira D.P.L., Ribeiro E., Barcelos C.A.Z. (2018). A variational approach to non-rigid image registration with Bregman divergences and multiple features. Pattern Recognit..

[8] Darkner S., Pai A., Liptrot M.G., Sporring J. (2018). Collocation for diffeomorphic deformations in medical image registration. IEEE Trans. Pattern Anal. Mach. Intell..

[9] Studholme C., Hill D., Hawkes D. (1999). An overlap invariant entropy measure of 3D medical image alignment. Pattern Recognit..

[10] Zhu X., Ding M., Huang T., Jin X., Zhang X. (2018). PCANet-based structural representation for nonrigid multimodal medical image registration. Sensors.

[11] Öfverstedt J., Lindblad J., Sladoje N. (2019). Fast and robust symmetric image registration based on distances combining intensity and spatial information. IEEE Trans. Image Process..

[12] Nie Z., Yang X. (2019). Deformable image registration using functions of bounded deformation. IEEE Trans. Med. Imaging.

[13] Rohlfing T. (2012). Image similarity and tissue overlaps as surrogates for image registration accuracy: Widely used but unreliable. IEEE Trans. Med. Imaging.

[14] Rueckert D., Clarkson M.J., Hill D.L.G., Hawkes D.J. Non-rigid registration using higher-order mutual information. Proceedings of the SPIE Medical Imaging.

[15] Pluim J.P., Maintz J.A., Viergever M.A. (2000). Image registration by maximization of combined mutual information and gradient information. IEEE Trans. Med. Imaging.

[16] Loeckx D., Slagmolen P., Maes F., Vandermeulen D., Suetens P. (2010). Nonrigid image registration using conditional mutual information. IEEE Trans. Med. Imaging.

[17] Wachinger C., Navab N. (2012). Entropy and Laplacian images: Structural representations for multi-modal registration. Med. Image Anal..

[18] Heinrich M.P., Jenkinson M., Bhushan M., Matin T., Gleeson F.V., Brady M., Schnabel J.A. (2012). MIND: Modality independent neighbourhood descriptor for multi-modal deformable registration. Med. Image Anal..

[19] Heinrich M.P., Jenkinson M., Papiez B.W., Brady S.M., Schnabel J.A. Towards realtime multimodal fusion for image-guided interventions using self-similarities. Proceedings of the 16th International Conference on Medical Image Computing and Computer-Assisted Intervention.

[20] Heinrich M.P., Jenkinson M., Brady S.M., Schnabel J.A. Globally optimal deformable registration on a minimum spanning tree using dense displacement sampling. Proceedings of the 15th International Conference on Medical Image Computing and Computer-Assisted Intervention.

[21] Zhu F., Ding M., Zhang X. (2016). Self-similarity inspired local descriptor for non-rigid multi- modal image registration. Inf. Sci..

[22] Piella G. (2014). Diffusion maps for multimodal registration. Sensors.

[23] Morales J.L., Nocedal J. (2011). Remark on “algorithm 778: L-BFGS-B: Fortran subroutines for large-scale bound constrained optimization”. ACM Trans. Math. Softw..

[24] Klein S., Staring M., Pluim J.P.W. (2007). Evaluation of optimization methods for nonrigid medical image registration using mutual information and B-splines. IEEE Trans. Image Process..

[25] Wachowiak M.P., Smolikova R., Zheng Y., Zurada J.M., Elmaghraby A.S. (2004). An approach to multimodal biomedical image registration utilizing particle swarm optimization. IEEE Trans. Evol. Comput..

[26] Yang F., Ding M., Zhang X., Hou W., Zhong C. (2015). Non-rigid multi-modal medical image registration by combining L-BFGS-B with cat swarm optimization. Inf. Sci..

[27] Camara O., Delso G., Colliot O., Moreno-Ingelmo A., Bloch I. (2007). Explicit incorporation of prior anatomical information into a nonrigid registration of thoracic and abdominal CT and 18-FDG whole-body emission PET images. IEEE Trans. Med. Imaging.

[28] Tang S., Fan Y., Wu G., Kim M., Shen D. (2009). RABBIT: Rapid alignment of brains by building intermediate templates. NeuroImage.

[29] Zacharaki E.I., Hogea C.S., Shen D., Biros G., Davatzikos C. (2009). Non-diffeomorphic registration of brain tumor images by simulating tissue loss and tumor growth. Neuroimage.

[30] Brun C.C., Leporé N., Pennec X., Chou Y.Y., Lee A.D., De Zubicaray G., Thompson P.M. (2011). A nonconservative lagrangian framework for statistical fluid registration—Safira. IEEE Trans. Med. Imaging.

[31] Foi A., Boracchi G. (2016). Foveated nonlocal self-similarity. Int. J. Comput. Vis..

[32] Glocker B., Komodakis N., Tziritas G., Navab N., Paragios N. (2008). Dense image registration through MRFs and efficient linear programming. Med. Image Anal..

[33] Komodakis N., Tziritas G., Paragios N. (2008). Performance vs computational efficiency for optimizing single and dynamic MRFs: Setting the state of the art with primal-dual strategies. Comput. Vis. Image Underst..

[34] Brainweb. http://www.bic.mni.mcgill.ca/brainweb/.

[35] NA-MIC Data. http://na-mic.org/Wiki/index.php/Projects:RegistrationLibrary:RegLib_C47..

[36] NA-MIC Data. http://na-mic.org/Wiki/index.php/Projects:RegistrationLibrary:.RegLib_C20.

[37] Retrospective Image Registration Evaluation Project. https://www.insight-journal.org/rire/.

[38] Mercier L., Del Maestro R., Petrecca K., Araujo D., Haegelen C., Collins D. (2012). Online database of clinical MR and ultrasound images of brain tumors. Med. Phys..

[39] BITE Database. http://nist.mni.mcgill.ca/?page_id=248.

[40] Maurer C.R., Fitzpatrick J.M., Wang M.Y., Galloway R.L., Maciunas R.J., Allen G.S. (1997). Registration of head volume images using implantable fiducial markers. IEEE Trans. Med. Imaging.

[41] Wang C.W., Chen H.C. (2013). Improved image alignment method in application to X-ray images and biological images. Bioinformatics.

[42] West J., Fitzpatrick J.M., Wang M.Y., Dawant B.M., Maurer C.R., Kessler R.M., Maciunas R.J., Barillot C., Lemoine D., Collignon A. (1997). Comparison and evaluation of retrospective intermodality brain image registration techniques. J. Comput. Assist. Tomogr..

[43] Rivaz H., Chen S., Collins D.L. (2015). Automatic deformable MR-ultrasound registration for image- guided neurosurgery. IEEE Trans. Med. Imaging.

